# A call to action: more parent–child interaction research within daily routines!

**DOI:** 10.1093/jdsade/enaf057

**Published:** 2025-12-17

**Authors:** Martina Curtin, Evelien Dirks, Amy Szarkowski

**Affiliations:** Homerton Healthcare NHS Foundation Trust, London, United Kingdom; Language and Communication Science, City, University of London, London, United Kingdom; Tranzo, Tilburg University, Tilburg, the Netherlands; NSDSK Amsterdam, the Netherlands; Institute for Community Inclusion, University of Massachusetts Boston, Boston, MA, United States

## Abstract

Parent–child interaction (PCI) is known to be a suportive predictor of child developmental outcomes. PCI is a 2-way, connected exchange where, particularly in infancy, the child is the agent of the interaction, initiating with eye gaze, action, emotion, and/or language, and the parent adapts their communication and interactional style in order to attune to and respond to their child. The research objectives of this study were informed by a group of hearing parents of deaf and hard-of-hearing (DHH) children. Whereas much of the literature examining PCI involves observations of parents/caregivers interacting with their children during play or book reading, these parents noted that PCI happens throughout the day; therefore, guidance and support to promote PCI during daily routines (i.e., repeated routines in care, hygiene, and nutrition) would *also* be useful. A systematic review aimed to identify and synthesize all academic papers assessing PCI with DHH children aged 0–3 within daily routine activities at home (i.e., activities outside of play or book reading). Severely limited results led to a 4-point Call to Action for researchers in the field: (1) examine PCI in daily routines; (2) capture PCI using video; (3) recruit diverse participant groups; and (4) report explicitly.

Parent–child interaction (PCI) is a well-researched, multi-faceted phenomenon present between every child and parent. It is an umbrella term that encompasses a range of child and parent behaviors that lead to a bidirectional, reciprocal relationship. For example, if the child is the agent of the interaction, they might involve their parent by pointing, using eye gaze, or initiating some other form of early communication. The parent then may attune to their child and respond in a way that acknowledges the child’s interests, gaze, behavior, or language, such as moving a toy, using an accompanying sound, or a single word or sign that is child-directed in its delivery. Together, parents and children can contribute to mutually regulated turn-taking and social exchanges (Hoehl & Markova, 2018).

## Implications of PCI in families with children who are deaf or hard of hearing

Researchers have identified variability in PCI quality in the typical hearing population and have found associations with children’s language outcomes (Hoff & Laursen, 2019), cognition (Ramos et al., 2023), and social–emotional functioning (Stern et al., 2024). For families with children who are deaf or hard of hearing (DHH),^1^ PCI is of particular relevance.

Reciprocal interactions and “intuitive parenting” can, at times, be challenged when a child is identified as deaf^2^ (Traci & Koester, 2003). Differences in hearing status between parents and the DHH child can cause difficulties with gaining and maintaining the DHH child’s attention and ensuring that the DHH child has access to language. This can result in PCI that is more directive or structured by the parent, compared to hearing dyads. Many hearing parents benefit from family-centered early intervention (FCEI) that helps to adapt and enhance their intuitive parenting, to attain successful interactions with their DHH child (Dirks & Rieffe, 2019). It can also be true that DHH parents of DHH children can also benefit from receiving support for PCI. Parents’ engagement, responsiveness, and linguistic input may be particularly important for DHH children compared to hearing children (Pressman et al., 1998; Stevenson et al., 2015).

Caregivers frequently modify their parenting style as a result of having a young child who is DHH and describe needing to take an active role in filtering interactions between the child and the child’s environment (Vukkadala et al., 2019). Parents with DHH children who have co-occurring conditions or medical complexities report emotional and physical “drain” that can make them less available for positive interactions with their DHH child (Whicker et al., 2019). Thus, while the literature on PCI among typically developing children and their parents can inform and underpin understanding of PCI in dyads involving DHH children, there is a need to explore the unique aspects and features of these dynamics in this population.

### Notable parent behaviors important for interactions with DHH children

Many parent behaviors identified within PCI studies with young DHH children aged 0–3 years (Curtin et al., 2021, 2023; Lammertink et al., 2022) are the same as those described in typical hearing parent–hearing child dyads (Gridley et al., 2019; Roberts & Kaiser, 2011). Parent behaviors *specifically* relevant for PCI with DHH children focus on gaining and maintaining the child’s attention (Barker et al., 2009; Dirks & Rieffe, 2019; Gale & Schick, 2009; Lammertink et al., 2022; Loots et al., 2005; Morgan et al., 2021; Wille et al., 2019) and promoting access to language, such as being face to face, adjusting positioning to be near to amplification devices, or emphasizing child-directed speech or sign (Curtin et al., 2021; Houston et al., 2003; Mayberry & Squires, 2006; Wang et al., 2017). Scholars have found that PCI predicts how well a DHH child develops language skills (Holzinger et al., 2020; Pressman et al. 1998; Quittner et al., 2013). The parent of a DHH child needs to show synchronicity to a DHH child’s attention to remain jointly engaged (Majorano et al., 2021). The same behavior in a hearing child–hearing parent dyad may reflect potentially different dynamics. A hearing child can center their gaze on objects and still benefit from the auditory language input provided by their parent during supported and/or coordinated joint engagement. The parent of a hearing child does not necessarily need to consider whether their child-directed language is accessible or perceivable. In DHH child–hearing parent dyads, establishing and maintaining joint engagement is different because the parent must be very sensitive to the DHH child’s visual and auditory attention, providing contingent, synchronous language input when their child is looking and/or listening (Houston, 2022). For DHH children using spoken language, speech perception in a noisy listening environment adds another layer of difficulty in terms of attended-to language; indeed, speech in noise has been deemed inaccessible (Majorano et al., 2021). For DHH children developing signed languages, a parent must ensure the child perceives the sign, any visual referents (e.g., eye gaze, pointing), and the real object sequentially, all through their visual channel of attention (Lieberman et al., 2022).

### The assessment of PCI with DHH children

Assessment of PCI among young DHH and hearing children predominantly involves two main activities (Curtin et al., 2021; Holme et al., 2023). Play is the most frequently selected activity for PCI research; often, theories are being tested or interventions are being trialed, and controls are established (i.e., standardizing the setting, toys, and length of the observations) in an effort to improve the rigor of the study and the validity of the resultant findings. The second most popular activity in research examining PCI in both hearing and DHH populations is shared book reading (DesJardin et al., 2023; Dirks & Wauters, 2018; Ece Demir-Lira et al., 2019; Gilkerson et al., 2017; Holme et al., 2023; Wauters & Dirks, 2017; Wauters & Dirks, 2024). The context of storybook reading is often used in research with DHH children due to its relation to language development, given the importance and potential risks associated with language development among children who are DHH.

### Daily routines and the assessment of PCI

While acknowledging the importance of PCI during play and book reading, it is also important to explore PCI through daily routines, which have received much less attention within the PCI literature. In the current paper, daily routines refer to childcare activities that take place regularly within home settings, including sleep, hygiene, or nutrition (i.e., nappy changing, dressing, bathing, breast/bottle feeding, eating or preparing food, cleaning/tidying up, preparing for sleep). Parents engage in these activities every day, whereas play and book reading may be “family routines” that occur on some or most days. The context of daily routines provides a meaningful and natural setting for examining interactions between parent and child. Daily routines offer consistent opportunities for parents and children to engage in communication and bonding. These routines create familiar environments where interactions occur regularly, making them ideal for observing the dynamics of parent–child interactions and understanding how these interactions support child development (Selman & Dilworth-Bart, 2024). Daily routines consist of repeated activities shared by two or more household members, offering structure and organization to daily family life (Fiese & Everhart, 2008). Daily routines are regular, established patterns of activities that bring order and efficiency to everyday life. The predictability of daily routines helps children to understand the sequence of events and what is expected from them (Nelson, 1985; Tamis-LeMonda et al., 2018). This can reduce uncertainty, anxiety, and stress, allowing children to focus on exploration and learning.

Recent research involving hearing parent/hearing child dyads suggests that PCI during play and mealtime is quite similar, namely, facets of joint engagement, mutual gaze, parents’ naming of objects, and amount of parent talk (Schroer et al., 2024). Alternatively, examining PCI during daily routines could show promise in advancing understanding of salient aspects of PCI and the socio-cognitive approach of language acquisition. Daily routines provide a multi-modal springboard for word learning and concept development within real life (Tomasello, 2001). As Tamis-LeMonda et al. (2019) explain from their video-based observational study of hearing dyads in the home, infants map words to the people, actions, and objects of familiar events. They learn words within regularly repeated, multi-sensory environments (e.g., they hear about body parts while in the bath, getting wet, and smelling soap; they also learn about utensils, food, and the related verbs while seeing caregivers prepare meals, smelling food, and tasting items. This usage-based context explains why children’s first words are often concrete (Hoff, 2010; Snow, 1983; Tomasello, 2005). Language input for young children typically includes often-repeated terms connected to the child’s environment (Hills et al., 2009; Wiemer-Hastings et al., 2004).

Recent studies with DHH infants have sought to capture interactions throughout the whole day (e.g., Brock & Bass-Ringdahl, 2021), although these have small sample sizes and embrace all-day, audio-only recording software (e.g., LENA Pro software). A recent study from Smolen et al. (2021) focused on PCI during dinnertime, albeit with older DHH children aged 3–6 years. Researchers extracted dinner time segments from all-day, audio-only recordings of 37 children, each 20 min in length, using LENA Pro software. The audio was transcribed and then coded for parents’ use of high- and low-level language facilitation techniques. Children’s receptive vocabulary and understanding of basic concepts were also assessed. Results suggested that during dinner time, “lower-level techniques” were employed such as directives (i.e., “Look, a bird!” or “Don’t touch that”) and close-ended questions (i.e., “What’s that?” or “Do you want a cookie?”). Importantly, positive correlations were found between the spoken English receptive vocabulary of children who are DHH and their parent’s use of open-ended language facilitation, explicit vocabulary instruction, and imitation. The study team concluded that for some families, dinner time does not necessarily serve as a language-learning opportunity.

Muller et al. (2024) used LENA Pro software to examine the quantity and quality of parental talk during both regular and focused interactions during the day with children who are DHH. The scholars defined “focused” as a high number of conversational turns between parent and child and “regular” as interactions with fewer turns. They also examined what kind of activities occurred during regular and focused interactions. The top three activities during focused interactions were play (25%), care (21%), and storybook reading (17%); the top three activities during regular interactions were play (29%), mealtime (17%), and care (13%). Daily routine activities feature in both types of interactions, and therefore could be seen as language learning opportunities. This may be particularly important for families where play and book reading feature less within their family routines.

Each of the studies mentioned above (Brock & Bass-Ringdahl, 2021; Muller et al., 2024; Smolen et al., 2021) used LENA software to audio record the whole day. LENA software has contributed relevant and important data to the field of PCI. It provides a unique way of recording a child’s daily life without being highly intrusive and arguably provides more naturalistic data than a 10-min video recording. However, audio-only analyses miss the visual elements of PCI, which are particularly important with DHH infants. Even when the dominant language(s) used within families are spoken language(s), important features of PCI (such as facial expression, gaze, eye contact, affect, and gesture) are not captured through audio-only data collection methods. DHH children who use signed language(s) or who use some combination of spoken language and signed language are unable to be included, or, if they are included in studies that use audio-only recordings, salient aspects of their interactions with their caregivers will not be captured. To better understand essential aspects of PCI among dyads with children who are DHH, inclusion of the visual elements of connection and exchanges is *also* necessary. Video captured PCI is more inclusive, and findings may better reflect practice; early intervention providers and practitioners observe PCI between hearing and DHH dyads live, using visual and auditory channels to make sense of the PCI they observe (Curtin et al., 2023).

### Development of research questions with patient and public involvement

“Nothing about us, without us, is for us” (Rahman et al., 2022, p.1) explicitly demonstrates the moral and ethical rights that patients and carers *should* have in being involved in research that potentially impacts them and/or their families. PPI has been shown to improve the value of research by increasing its relevance to patients, ensuring that research conducted provides patient benefit, increasing the accountability of researchers and the transparency of research, and enhancing dissemination beyond academia, making relevant research findings more broadly available (Ghate, 2018; Greenhalgh et al., 2019; Tembo et al., 2021). Although the term “patients” may not be as directly applicable, the sentiment remains; in studies exploring experiences of parents of DHH children, parents should be included.

The research questions of this systematic review were influenced by discussions with the parent partner group of the Early Parent Interaction with Deaf children project (see Curtin et al., 2021, 2023, 2024, 2024). These were 14 U.K.-based hearing parents whose DHH children were different ages (ranging from 12 months to 14 years old), had differing levels of identified deafness, and collectively used a wide range of spoken and signed languages.

A panel of 83 international academic and professional experts was invited to form a consensus on the content and recommended procedure of a forthcoming PCI tool by voting on a series of statements (Curtin et al., 2024). One of the statements included in the aforementioned study, generated from the PPI parent partner group, suggested “as well as observing interaction in play, professionals could sample interactions within daily routines (e.g., mealtimes, dressing) where parents are willing” Curtin et al., 2024; p.16). A total of 78% of the expert panelists agreed with this statement in the study; however, the statement needed to achieve 80% agreement to be included. When this close-to-consensus statement was discussed with the parent partner group, strong opinions regarding the need to include this statement were shared. Some parents felt that, particularly in busy households, an assessment of PCI during daily routines could be a welcome suggestion (Curtin et al., 2025), as discussing behaviors in these contexts may result in easier-to-implement changes in a parent’s interactive behaviors. In wider research contexts, embedding support into daily routines has proven beneficial for families, particularly in generalizing skills (Novak, 2011). While a recent systematic review uncovered a range of parent behaviors observed in PCI during play-based contexts (Curtin et al., 2021), the parent partner group pondered whether any research was being carried out that examined different or additional parent behaviors during PCI in the context of daily routines, given that it is likely that daily routines (as defined on page 5) makeup a great proportion of family time. Therefore, a systematic review was deemed the most appropriate initial step for examining this parent-informed question regarding PCI within families with children who are DHH. It was hypothesized that few studies in this field would exist.

The research questions for this systematic review on daily routines are:

1. What is the range of parent behaviors assessed or described in parent–child interaction (PCI) among dyads with DHH children aged 0–3 years?
2. How are parent–child interaction behaviors assessed? (e.g., What are the tools used?)
3. Which daily routine contexts involving parents and their DHH children aged 0–3 years have been explored?
4. How are parent behaviors, child behaviors, parent–child interactions, and parent–child routines related to child development outcomes? (i.e., Did the PCI studies with DHH children explore associations between PCI behaviors and child outcomes? If so, what associations were reported?)

## Methods

This systematic review was conducted following guidance from the Cochrane Handbook for Systematic Reviews (Cumpston et al., 2019). The Preferred Reporting Items for Systematic Reviews and Meta-Analyses (PRISMA) Statement (Page et al., 2021) was used to ensure robust reporting. The research team developed and approved the review protocol, which was then externally reviewed and made available by the team at PROSPERO (CRD42023432822).

### Selection criteria

For this work, all peer-reviewed, published studies available in English that included DHH infants aged 0–3 years with any level of identified deafness, any amplification (or none), and any communication modality were included. Included papers had to investigate interactions between the DHH child and the child’s parent within everyday routines within the home. Parents could be hearing or DHH.

All study types (quantitative, qualitative, or mixed methods) and any research designs (randomized control trial, intervention, or observational studies) were included. PCI assessment had to be objectively measured in the research studies, through nonvalidated and/or validated measures. To be included, papers were required to report results on parent behaviors as well as some aspects of the parent–child interaction. Papers were excluded if they analyzed PCI during free play, structured play, or book reading (as explained on pages 4 and 9, these are already well-researched contexts). Papers were also excluded if they used subjective data (i.e., parent self-report) to analyze PCI or only reported on child behaviors within PCI.

### Search strategy

The first author searched the following eight databases on 6 June 2023: Medline, PsycINFO, CINAHL, Communication Source, Cochrane Databases, Embase, Web of Science, and Scopus, through two platforms, Ovid and EBSCOhost. Two types of searches were conducted. Search One, which used synonyms for “deaf,” “infant,” “parent,” and “interaction” had a date limit of June 1, 2020 to present. Prior to this date, search and screening from a previously published systematic review was used, as this already captured all DHH-related PCI studies published prior to that date (Curtin et al., 2021). In Search Two, synonyms for “deaf,” “infant,” “parent,” and “daily routines” were used to ensure that papers related to daily routines previous to June 2020 were not missed. There were no limits or date restrictions to Search Two. Please see Appendix A for the full search strategy.

### Selection process

Covidence software was used in the review and data collection process. All search results were uploaded, and duplicates were automatically removed. An additional 77 papers were included from a previous systematic review (Curtin et al., 2021); they had previously been excluded from that review, which focused on PCI during play, because they were determined not to meet the inclusion criteria because they were “not play.” Therefore, the authors queried if any of these were daily routine activities. As an initial trial, five papers were reviewed independently by authors 1 and 3; conflicts that arose were discussed, and the researchers’ framing of the inclusionary criteria was tightened. Each paper was then independently screened based on the article title and abstract. Authors 1 and 3 met 97% agreement (*k* = 0.83) at this stage.

Full texts were retrieved for the articles that met the inclusion criteria. Each paper was independently reviewed by Authors 1 and 3. Discrepancies were resolved every 1–2 weeks. Authors met 91% agreement (*k* = 0.66).

### Data collection process

Each included paper in the review was independently extracted and reviewed by the first author and at least one other author. All authors were involved in meetings to gain consensus, check discrepancies, and make final decisions.

### Data items

The extraction form (Appendix B) was written by the first author, then reviewed and amended by the other authors, before being added to Covidence.

To answer Research Questions 1, 2, and 3, the authors examined the research to determine: (a) the main outcome variables described; (b) information about the content of the PCI assessment (including descriptions of behaviors investigated and how the interactions were assessed—that is, where, for how long and how often); and (c) the means by which PCI was measured (i.e., whether coding systems or scales were used and who completed them, and what information was provided regarding reliability). The results of the assessments were also collected to answer research Question 4.

Other variables captured in the systematic review included: study characteristics [e.g., country of study, research design, conflicts declared); participant characteristics (i.e., child age, levels of identified deafness, amplification used, communication mode(s) used by the child and the parent, parent age, social economic status, parent education level]; intervention characteristics if any (e.g., intervention name, delivery, dose); and confounders and limitations identified by the authors. Missing information was labeled as “not reported.”

### Risk of bias assessment

The Joanna Briggs Institute Critical Appraisal Checklist for Cross-Sectional Studies (Moola et al., 2020) was used as the risk of bias assessment. No adaptations were made. The tool includes guidance for how to assess risk of bias in research. Through the answering of eight questions, reviewers decide on an overall risk of bias judgment. Similar to the process employed for data extraction, the risk of bias for each of the studies was independently reviewed by the first author and at least one other author, with differences resolved in regular meetings.

### Synthesis (preparation and approach)

Extracted data were exported into Excel from Covidence, and a summary table of the included papers was created. While the aim was to use a narrative synthesis approach, using guidance from Rodgers et al. (2009), Popay et al. (2006), and Campbell et al. (2019), this was not required for reasons stated below.

## Results

**Figure 1:**
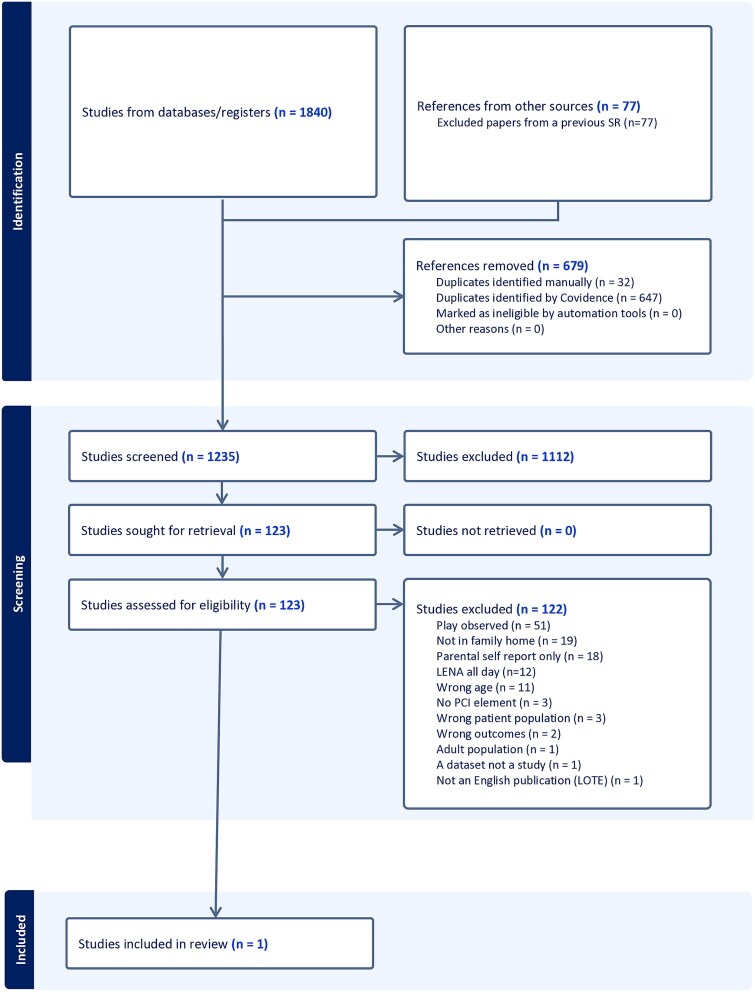
PRISMA Table [produced by Covidence Systematic Review Software (2024), Veritas Health Innovation, Melbourne, Australia].

In total, 1840 papers were identified and included in the selection process. Following title and abstract screening, 123 papers were retrieved for the full-text review. After in-depth reading, one paper met the inclusion criteria for this review. See PRISMA (Page et al., 2021) flowchart from Covidence (Figure 1) for more details. Table 1 outlines the key features of this paper in relation to the research questions for the present systematic review (in short, the parent behaviors assessed, methods of assessment, context assessed, and whether child language or any other developmental outcomes were assessed). However, as only one paper was included (described below in depth), we acknowledge that our research questions cannot be answered.

**Table 1:** Included paper continued (NR = not reported)

First author, year, country	Study design	All aged 0–3?	Total dyads (*n*)	Hearing dyads included? (*n*)	Hearing devices used	Languages used by the child	Child exposed to sign?	Additional needs	Ethnicity	Ses	Parent education level
Vanormelingen et al., 2015, Belgium	Longitudinal between groups, observational	Yes	20	Yes (10)	CI	Dutch	NR	NR	NR	Mid to High SES	NR
**First author, year**	**Child assessed in any way? How?**			**Parent behaviors assessed**		**PCI measure**	**Context**		**Are video recordings used?**		
Vanormelingen et al., 2015	Child vocalizations, using a novel measure			1) Whether the parent responded (or not) to the child’s vocalization 2) Whether the parent uses child utterance within response or not.		Coding	Spontaneous interactions		Yes		

The included paper was published in 2015 by Vanormelingen and colleagues (see Table 1). This was a repeated-measures, cross-sectional, between-groups, video-based, observational study of PCI with 10 hearing children and 10 DHH children who had received cochlear implants (CIs). Data had been extracted from a child language corpus. All families were monolingual and Dutch speaking. It was assumed the families were residing in Belgium, although the location in which the study was conducted was not reported. Parents’ socioeconomic status was described as “middle to high.” Exposure to a signed language, presence of additional needs, ethnicities of the families, and parental education levels were not reported. Descriptions of family and child demographics were limited and inclusion/exclusion criteria were not included (possibly because data were extracted from a corpus). Thus, the authors of the current research rated the paper’s methodological quality in terms of potential bias, as “serious” as assessed using the JBI Tool (see risk of bias assessment, Moola et al., 2020). The paper described observing “spontaneous interactions”; therefore, it is not clear which activities associated with PCI were being monitored. The authorship team debated whether to include this paper as it is highly likely that across the multiple recordings of these dyads, play and shared book reading would have also been observed (again, the authors note the importance of those activities, yet sought to explore PCI in other contexts). In the end, it was included because there was no direct mention of play or book reading, and the interactions assessed were described as being between the mother and the child.

The parent behaviors analyzed in the study by Vanormelingen et al. (2015) included whether a mother responded to the child’s vocalizations/utterances, what type of child utterance the mothers responded to, and how the mothers responded. Video recordings of parent–child interaction, between 50 and 120 min in length, were taken monthly at the family home, from 6 to 24 months for the hearing children, and from 1 to 30 months post-CI activation for the DHH children. Twenty minutes of each recording, where the child was most vocally active, were transcribed. As mentioned, the type of activity observed appears vague, described as “spontaneous interactions.” This study did not attempt to suggest how these parent behaviors impacted child vocalizations.

There were four other papers that almost met the inclusionary criteria for the current systematic review. These were Cheskin (1981), Harrigan and Nikolopoulos (2002), Adi-Bensaid and Greenstein (2020), and Odijk and Gillis (2021). Except Cheskin, the remaining three studies used video recordings at home to analyze PCI. Harrigan and Nikolopoulos (2002) instructed families to “choose the activity”, Odijk and Gillis (2021) requested that “parents interact normally,” and Adi-Bensaid and Greenstein (2020) captured “natural activities.” While all four papers included some descriptions of daily routines such as baking cakes, having a meal, bathing, and dressing, they also specifically mentioned play. PCI captured in daily routines were not analyzed or reported separately to PCI captured in play in these studies; therefore, all four articles were excluded. Similarly, 12 papers (see Table 2) used LENA Pro software as the sole method of capturing whole-day recordings. While daily routines would have been captured, it is very likely that play and book reading would be activities also included in these data and this differentiation/disaggregation was made not in the analysis or results. Most importantly, LENA Pro Software captures *all* caregiver and adult voices, meaning there can be less specificity on one parent’s interaction with their DHH child. Because these 12 papers likely included play and book reading, and most papers included all adults in the child’s environment (not just parents), they were excluded.

**Table 2:** Papers included DHH children under 3, and investigated facets of PCI (linguistic input), yet excluded papers on the grounds of all-day recordings and therefore likely to include play and book reading, and for some, other adults

Ambrose, S. E., Vandam, M., & Moeller, M. P. (2014). Linguistic input, electronic media, and communication outcomes of toddlers with hearing loss. Ear and Hearing (01960202), 35(2), 139–147, doi:10.1097/AUD.0b013e3182a76768 Arjmandi, M. K., Houston, D., & Dilley, L. C. (2022). Variability in quantity and quality of early linguistic experience in children with cochlear implants: Evidence from analysis of natural auditory environments. Ear and Hearing, 43(2), 685–698. DOI: 10.1097/AUD.0000000000001136 Benítez-Barrera, C. R., Angley, G. P., & Tharpe, A. M. (2018). Remote microphone system use at home: Impact on caregiver talk. Journal of Speech, Language & Hearing Research, 61(2), 399–409, doi:10.1044/2017_JSLHR-H-17-0168 Brock, A. S., & Bass-Ringdahl, S. M. (2021). Facilitative language techniques used in the home by caregivers of young children who are deaf or hard of hearing. Perspectives of the ASHA Special Interest Groups, 6(5), 1,137–1,145. DOI: 10.1044/2021_PERSP-20-00297 Ganek, H., Nixon, S., Smyth, R., & Eriks-Brophy, A. (2019). A Cross-cultural mixed methods investigation of language socialization practices. Journal of Deaf Studies & Deaf Education, 24(2), 128–141, doi:10.1093/deafed/eny037 Ganek, H., Smyth, R., Nixon, S., & Eriks-Brophy, A. (2018). Using the Language ENvironment Analysis (LENA) system to investigate cultural differences in conversational turn count. Journal of Speech, Language & Hearing Research, 61(9), 2,246–2,258, doi:10.1044/2018_JSLHR-L-17-0370 Kishida, Y., & Kemp, C. (2022). Improving Parents’ Interactions with Children with Hearing Loss Using Data-based Feedback, International Journal of Disability, Development and Education, 69:4, 1,216–1,234, DOI: 10.1080/1034912X.2020.1767761 Kristensen, N. M., Sundby, C. F., Hauge, M. N., & Lofkvist, U. (2020). Female caregivers talk more to 18–56-months-old children with and without hearing impairment than male caregivers measured with LENA (TM): A cross-sectional pilot study. International Journal of Pediatric Otorhinolaryngology, 130, 9, doi:10.1016/j.ijporl.2019.109809 Kondaurova, M. V., Zheng, Q., VanDam, M., & Kinney, K. (2022). Vocal turn-taking in families with children with and without hearing loss. Ear and Hearing, 43(3), 883–898. doi:10.1097/AUD.0000000000001135. Saetre-Turner, M., Williams, C., & Quail, M. (2015). Caregiver-child interaction in children who are deaf or hard of hearing and children who are normally hearing: Preliminary data. Journal of Clinical Practice in Speech-Language Pathology, 17(3). Thompson, E. C., Benítez-Barrera, C. R., Angley, G. P., Woynaroski, T., & Tharpe, A. M. (2020). Remote microphone system use in the homes of children with hearing loss: Impact on caregiver communication and child vocalizations. Journal of Speech, Language & Hearing Research, 63(2), 633–642, doi:10.1044/2019_JSLHR-19-00197 Vandam, M., Ambrose, S. E., & Moeller, M. P. (2012). Quantity of parental language in the home environments of hard-of-hearing 2-year-olds. Journal of Deaf Studies & Deaf Education, 17(4), 402–420, doi:10.1093/deafed/ens025

## Discussion

As correctly hypothesized, there is a dearth of research that examines PCI within daily routines involving children who are DHH and aged 0–3 years. A similar pattern for language-promoting activities in the hearing population also exists. In a review of 60 studies of PCI with hearing children aged 0–3 years (Holme et al., 2021), play and book reading were most frequently observed (31% and 27%, respectively) with daily routines observed less frequently (meal time 10% and personal care 5%). Similar to an existing study that examined PCI among parents and DHH children (Curtin et al., 2021), most studies within the hearing population are with mothers (89%) who are Caucasian or White European/American (85%) (Holme et al., 2021).

Positive PCI that is used across a variety of activity contexts has been associated with and/or shown to predict children’s vocabulary development ( Schroer et al., 2024; Tamis-LeMonda et al., 2019; Weizman & Snow, 2001). In addition to language development, daily routines are associated with positive developmental outcomes for all children. For example, cognitive control (Semenov & Zelazo, 2019) and emotional regulation (Chen, 2017). Research also suggests the protective power of routines in challenging environments (Bater & Jordan, 2017; Ren & Fan, 2019; Selman et al., 2024). Thus, a focus on PCI during daily routines offers substantial, cross-developmental benefits for DHH children and families.

This paper was generated as a response to concerns raised by a motivated and engaged group of parents of DHH children who shared their knowledge and lived experience on factors that can facilitate parent-implemented therapies, as well as factors that can become barriers to success. While all parents appreciated the importance of child play and book reading, parents expressed strong beliefs that evidence-based guidance on PCI during daily routines should *also* be on offer. Interventions that focus on PCI are, in the main, implemented by parents with guidance from trained practitioners (i.e., speech and language therapists, early interventionists, and/or teachers of DHH children). Parents might be more inclined to implement strategies for enhancing PCI, if doing so did not require them to build additional activities into their schedules. Parents articulated that being greater informed and being more intentional about engaging in PCI during everyday activities could help with the naturalness of interacting with their DHH child. Further, parents indicated a belief that centering positive PCI in all activity contexts may also remove guilt, bad feelings, and judgments among busy families, large families, and culturally diverse families (e.g., “We had no time for parent-supported play today, but that’s okay, we implemented the strategies at bath time, at nappy time, and during snack time instead”). When requests to carry out caregiver-led interventions are not met, parents can feel a sense of burden, overwhelm, and perhaps disengagement with sessions (Thomas et al., 2018), which could impact child outcomes. The authorship team acknowledges the benefits that play and book reading can bring to a child, their parents, and the shared relationship; yet, the researchers and parent panel that informed this research propose that daily routines should be considered as an activity for early intervention providers and practitioners to observe and support, *as well as* play and book reading.

In 2024, an international panel of experts published the Family-Centred Early Intervention Deaf and Hard of Hearing (FCEI-DHH) Call to Action (Szarkowski et al., 2024). One of the many recommendations within the FCEI-DHH document called for research that informs and advances Early Intervention and improves support for DHH children and their families. In response to the aforementioned FCEI-DHH Call to Action and in an effort to extend the field of PCI research to include families and their children who are DHH, we urge investigators to conduct research that addresses the following four points:

### Observe PCI within daily routines

“Everyday activities contain abundantly rich, multi-modal cues to word meaning” (Tamis-LeMonda et al., 2019, p. 2135). All families engage in, and regularly repeat, daily routines. To date, research examining PCI within daily routines is limited. More research is needed to understand the reciprocal, dynamic influences of child behaviors and parent behaviors on each other and on the relationship. For instance, there is a need to understand how daily routines support bonding, closeness, shared joy, affect, and touch, as well as outcomes in language, socioemotional, and overall development.

As encouraged by the FCEI-DHH special issue (Szarkowski et al., 2024), research should inform and advance support given to DHH children and their families. Research mirroring “real life” would be useful for our field and would align with calls for more ecological validity in child development studies (Coppens & Coppinger, 2023; Shamay-Tsoory & Mendelsohn, 2019). As we have seen in the case of Smolen et al. (2021), which examined mealtime specifically, and with Dirks (2025), which focused on diaper changing, it may be useful to look at PCI within a single daily routine context. This would aid comparison within the observations and across different studies if similar observational tools/coding systems were used. We acknowledge ethical approval and orchestrating studies may take more time, but they remain possible.

Professionals and researchers alike who support DHH children and their families would benefit from investigations of whether PCI between parents and their DHH children varies across different daily routine activities, and whether any additional “parent behaviors” are pertinent to raising a child who is DHH. Explorations of PCI during daily activities can yield valuable information such as: (a) what parent behaviors correlate with child outcomes across cultures and linguistic groups; (b) which daily routine contexts tend to be more parent-led and which provide more opportunities for parents and children to engage in more mutual exchanges (this finding could help practitioners select the daily routines that are best to observe, support and offer guidance for families); (c) whether it is acceptable or feasible for researchers to observe and/or professionals to offer support to foster PCI to families of DHH children during daily routines; and (d) whether, for those DHH children who wear assistive hearing devices, the daily routines of bath time and bedtime change when hearing equipment is off.

Further, we return to our parent partners’ request for this research to be conducted. As well as honoring their wishes in terms of this study’s research objectives, we also appreciate that there is a large body of evidence behind identifying and building on parents’ intuitive interaction behaviours and their already existing connections with their child, for example, strengths-based programmes such as Video Interaction Guidance (Kennedy et al., 2017; Kennedy & Underdown, 2017) and the Triple P Positive Parenting Program (Pourmohamadreza-Tajrishi et al., 2015; Proctor & Brestan-Knight, 2016; Stenason et al., 2020).

As mentioned, not every family can regularly prioritize play and book reading; thus, further research of PCI with daily routines across cultures, and via signed and spoken languages, can enhance the support and guidance offered to families with children who are DHH, ensuring effective, empowering, family-centered, strengths-based supports are implemented across national and global systems.

### Use video to capture PCI

Video recordings enable the capture of complex, multi-layered, naturalistic parent–child interaction (Lam-Cassettari et al., 2015; Wadnerkar Kamble et al., 2020). With video-based data collection, a range of multi-modal (and often simultaneous) parent and child behaviors can be documented. Capturing information with video provides opportunities for replay, micro-analysis, and frame-by-frame coding. According to an international consensus group on this topic, video recording of at least one instance of PCI is recommended to accurately capture and use to facilitate reflective conversations between parents and practitioners (Curtin et al., 2024). Given the agreement among professionals on the importance of video in enhancing and promoting PCI practice, the use of video in research must underpin that guidance.

In the current systematic review, at least 12 papers used LENA language processors to capture all-day, audio-only recordings of spoken language between DHH children and the adults with whom they interact. Capturing spoken language input and exposure, such as with the LENA language processor, can provide highly useful information that can serve an important purpose. Yet, we reiterate that audio-only recordings capture the adult’s and child’s spoken language. We do not see the nonverbal or visual elements of PCI that are particularly relevant for DHH children. Therefore, it is imperative that researchers embrace the data-rich opportunities that video can afford in PCI research so that both visual and aural aspects are included. We acknowledge that our Call to Action encourages researchers to record families in their homes during their private routines. This requires thorough planning and may, admittedly, be met with some resistance. We refer readers to best practice guidance on collecting long-form recordings from Cychosz and Cristia (2022). Interestingly, in a systematic review of 59 PCI papers with hearing children (Holme et al., 2021), the most frequently used method was video recordings (66% of studies), with fewer studies using audio-only (18%). Whereas in the field of PCI with DHH children, studies are using audio-only recording devices more frequently.

### Better inclusion

The population of DHH children and their families are diverse (van der Zee & Dirks, 2022). Studies of PCI with DHH children need to recruit more diverse samples, extending our understanding of how PCI can vary among a variety of caregivers including fathers and/or grandparents, with families from low-income backgrounds, with global minority populations (residing inside and outside of WEIRD^3^ countries), and within LGBTQIA+ families. The strengths, needs, perspectives, and outcomes of specific groups remain invisible when they are not participants in research or when they are included, yet not disaggregated in the analysis (Petkovic et al., 2020; Phillips & Hamberg, 2016).

There is a Westernized skew across all language acquisition research. In their review of speech perception, Singh et al. (2022) found only 10% of published studies focused on infants learning Asian languages, despite 60% of the global population residing in Asia. Studies on speech perception in Africa and South America were almost entirely absent. In a broader review of various topics within language acquisition over the last 45 years, Kidd and Garcia (2022) reported that 87% of the articles had authors based in North America or Europe. Family-centered early intervention for DHH children (FCEI-DHH), too, along with the policies and practices it has promoted, has been based on this partial evidence (Szarkowski et al., 2024). Changes to policy and practice are often based on Western, White-dominant research, which can contribute to certain groups being underserved, experiencing a higher level of burdens in health and/or social care and widening health inequalities (Barnes, Newby,, & NIHR Research Design Service Equality, Diversity and Inclusion Working Group, 2024). Inclusion needs to be embedded into research design from the beginning to avoid the same, repeated, researcher-centric limitations. We note aspiring work in this area from the Trial Forge group (Shepherd et al., 2024).

### Better reporting

As well as including diverse and underserved groups in the research of DHH children, we advocate for robust reporting of parent and child participant demographics. Analysis of the protocols that captured data from the research papers that were reviewed for the current systematic review revealed markedly inconsistent and inadequate reporting of demographic information. For studies involving DHH children, relevant hearing-related and demographic information is needed, for both the parents and children involved.

For the child characteristics, we suggest including: age of the child at the time of the study; gender; co-occurring conditions; levels of identified deafness; age of identified deafness; use of amplification, and consistency of using amplification; birth order of the child; whether they attend Early Intervention or FCEI-DHH (and details on what that is, how often, whether it is informed by knowledge of DHH needs); whether they are enrolled in childcare (how often); and their language skills (which language(s), used with whom, and language and communication assessment results where appropriate).

For families, we suggest including the following characteristics: parental age, hearing status, gender, and sexual orientation; parental education (highest qualification), ethnicity, and socioeconomic status; and parent-to-child communication (see “Language Access Profiles” from Hall & De Anda, 2021). We refer readers to the document titled “Diversity And Inclusion Survey (DAISY) Question Guidance” from the Equality, Diversity and Inclusion in Science and Health Initiative (2022) for suggestions regarding sensitively collecting information about participants’ characteristics.

Finally, when studying PCI with DHH children, we encourage researchers to describe the constructs being examined explicitly and report which element(s) of PCI are being assessed. Researchers can also clearly indicate which interlocutor(s) behaviors they are assessing (i.e., the mutuality and connectedness of the interaction between parent and child, or whether the evaluation is solely on parents’ or children’s behaviors.

## Limitations

This systematic review only included peer-reviewed articles published in English due to the skillset of the authorship team.

## Conclusions

This paper aimed to collect and synthesize all the relevant papers that focused on parent–child interaction with DHH children aged 0–3 years during daily routines (such as nappy changing, bathing, eating meals, and tidying up). As hypothesized by the authors, very few papers focused on daily routines. Most studies of PCI observed interactions between the DHH child and the parent during play and book reading, and most collected data via day-long audio recordings that captured spoken language within the DHH child’s environment. This current systematic review of the literature was prompted by input from a group of parents of DHH children who indicated that evidence-based guidance on the assessment and analysis of PCI during daily routines should *also* be on offer during FCEI, as they argued that children’s personal care and nutrition are relevant to all families.

With a dearth of evidence exploring daily routines and PCI with DHH children, the systematic review’s results were inconclusive. The paper therefore concluded with a four-point Call to Action for researchers in the field: (1) describe PCI constructs in research judiciously and consider the use of the umbrella term “PCI”; (2) examine PCI in daily routines; (3) capture PCI involving DHH children using video; (4) recruit diverse participant groups; and (5) report participant demographics thoroughly.

Studies regarding how families with DHH children engage in PCI during daily routines will help to inform the field, offering further understanding of aspects of PCI that might be unique to DHH children. Research in this area can also contribute to the provision of recommendations for EI Providers in FCEI and other professionals involved in supporting families with young children who are DHH related to encouraging, commenting on, or working alongside parents to address PCI.

## Supplementary Material

Supplementary_File_1_-_Appendix_A_Search_Terms_enaf057

Supplementary_File_2_-_Appendix_B_Extraction_Form_enaf057

## Footnotes

1 While the authors always encourage honoring individuals’ identities and language use preferences, compromises must be made to ensure consistency and clarity of the message in written form. Members of Deaf Communities are highly variable in their preference for whether to capitalize “Deaf” or use “deaf” to be more inclusive in writing about diverse individuals (Pudans-Smith et al., 2019). In a desire to move away from the medically and audiologically focused terminology related to *hearing loss* (e.g., National Health Service, U.K., https://www.nhsinform.scot/illnesses-and-conditions/ears-nose-and-throat/hearing-loss/, and the American Academy of Audiology, U.S., https://www.audiology.org/consumers-and-patients/hearing-and-balance/), and in recognition that children born DHH do not have the experience of “loss of hearing” (see British Academy of Audiology, https://www.baaudiology.org/positive-terminology/ for discussion), the authors use “*deaf or hard of hearing (DHH)*” to signal broad inclusion of all (see Szarkowski et al., 2024b for discussion on global perceptions on this topic). While the term *deaf* is believed to be inclusive of all individuals with identified deafness in some contexts, elsewhere, that term is generally accepted to mean individuals with greater levels of identified deafness and is not believed to be inclusive of those with some auditory access. In their paper on political involvement, Espinoza et al. (2024) provide a nice description of intersectional aspects of identity for deaf individuals, which may be useful to interested readers.

2 The authors of the current paper reside in different countries, where preferred terminology differs, with specific reference to “*hearing levels*” versus “*identified levels of deafnes*s.” It is acknowledged that in some cultural contexts, the term *deafness* is less desirable and is perceived as a social construct that carries with it assumptions about the experience of being DHH (Obasi, 2008). Deaf identities are complex and influenced by cultural conceptualizations of being DHH as well as lived experiences of being DHH in specific contexts (Leigh, 2017). The authors have decided to use the term “i*dentified levels of deafness”* to refer to an individual’s audiological status, rather than “*hearing level*,*”* which still frames the deaf child audiologically, as partially hearing.

3 WEIRD was coined by Henrich et al. (2010) and refers to Western, Educated, Industrialized, Rich, and Democratic countries.
