# Supplementary material for: A call to action: more parent–child interaction research within daily routines!
Source: J Deaf Stud Deaf Educ. 2025 Dec 17;31(2):189–202. doi: 10.1093/jdsade/enaf057 (PMC13324741; doi:10.1093/jdsade/enaf057)
Supplement: Supplementary_File_1_-_Appendix_A_Search_Terms_enaf057 [file supplementary_file_1_-_appendix_a_search_terms_enaf057.docx]

**FIRST SEARCH**

'deaf’ OR ‘deaf*' OR 'hearing impair*' OR 'hearing loss' OR 'hard of hearing' OR ‘d/hh’ OR ‘dhh’ OR ‘cochlear implant’ OR ‘hearing aids’ OR ‘hearing disorders’ OR ‘hard-of-hearing’

deaf OR deaf* OR hearing impair* OR hearing loss OR hard of hearing OR d/hh OR dhh OR cochlear implant OR hearing aids OR hearing disorders OR hard-of-hearing

AND

child OR child* OR infant* OR baby or babies OR preschool OR nursery OR toddler

AND

‘parent’ OR 'parent*’ OR ‘caregiver’ OR ‘care giver’ OR ‘mother’ OR ‘father’ OR ‘parenting’

parent OR parent* OR caregiver OR care giver OR mother OR father OR parenting

AND

‘involvement' OR 'interaction' OR ‘engagement’ OR ‘child behav*’ OR ‘parent behav*’ OR 'parent communication' OR ‘parent engagement’ OR ‘child-directed interaction’ OR ‘facilitative communication’ OR ‘parent interaction characteristics’ OR ‘sensitivity’ OR ‘responsiv*’ OR ‘linguistic input’ OR ‘language input’ OR ‘relationship’ OR ‘communication support strategies’ OR ‘dyad’ OR ‘availability’ OR ‘intersubjectiv*’ OR ‘attention’ OR ‘attend’

Involvement OR interaction OR engagement OR child behav* OR parent behav* OR parent communication OR parent engagement OR child-directed interaction OR facilitative communication OR parent interaction characteristics OR sensitivity OR responsiv* OR linguistic input OR language input OR relationship OR communication support strategies OR dyad OR availability OR intersubjectiv* OR attention OR attend

**SECOND SEARCH**

'deaf’ OR ‘deaf*' OR 'hearing impair*' OR 'hearing loss' OR 'hard of hearing' OR ‘d/hh’ OR ‘dhh’ OR ‘cochlear implant’ OR ‘hearing aids’ OR ‘hearing disorders’ OR ‘hard-of-hearing’

deaf OR deaf* OR hearing impair* OR hearing loss OR hard of hearing OR d/hh OR dhh OR cochlear implant OR hearing aids OR hearing disorders OR hard-of-hearing

AND

Child OR child* OR infant* OR baby or babies OR preschool OR kindergarten OR nursery OR toddler

AND

‘parent’ OR 'parent*’ OR ‘caregiver’ OR ‘care giver’ OR ‘mother’ OR ‘father’

parent OR parent* OR caregiver OR care giver OR mother OR father

AND

daily routines OR routines OR everyday activities OR activities of daily living OR snack OR dress* OR nappy OR diaper OR eat OR feed OR meal OR bath OR wash OR schedule OR brush OR sleep OR nap OR bedtime OR cook OR clean
