# Supplementary material for: A call to action: more parent–child interaction research within daily routines!
Source: J Deaf Stud Deaf Educ. 2025 Dec 17;31(2):189–202. doi: 10.1093/jdsade/enaf057 (PMC13324741; doi:10.1093/jdsade/enaf057)
Supplement: Supplementary_File_2_-_Appendix_B_Extraction_Form_enaf057 [file supplementary_file_2_-_appendix_b_extraction_form_enaf057.docx]

**Data Extraction Form**

| **General:** |  | | |
| --- | --- | --- | --- |
| Publication year |  | | |
| Country of study |  | | |
| **Study characteristics:** |  | | |
| Study design | TICK BOX:   1. Randomised controlled trial 2. Non-randomised experimental study 3. Between-groups intervention study 4. Within group pre/post intervention study 5. Between groups, observational study 6. Within group, observational study 7. Qualitative research 8. Case series 9. Other | | |
| Inclusion criteria |  | | |
| Exclusion criteria |  | | |
| **Participants** |  | | |
| No. of dyads assessed |  | | |
| **Child characteristics** |  | | |
| Total sample size – children (n) |  | |  |
| Sample size: Deaf children |  | |  |
| Sample size: Hearing children |  | |  |
| Are all children included in this study 36m or under? | Y/N | |  |
| Age ranges of child in months |  | | |
| % of males (please do calculation, number only) |  | | |
| Participating groups | | 1. Cochlear Implant (CI) only 2. Hearing Aid (HA) only 3. Mix of CI and HA (as 1 group) 4. CI vs. HA (2 groups) 5. CI vs. normal hearing (2 groups) 6. HA vs. normal hearing (2 groups) 7. CI vs. HA vs. normal hearing (3 groups) 8. CI AND HA vs. normal hearing (2 groups) 9. Other |  |
| Types of deafness included: | 1. Unilaterally deaf 2. Bilaterally deaf 3. A mix of unilateral and bilateral deafness 4. Not repor | | |
| Degree of deafness: | Mild: 25-34 dB  Moderate: 35-49 dB  Severe: 65 - 79dB  Profound: 80 dB+   1. Mild 2. Moderate 3. Severe 4. Profound 5. Not reported | | |
| When deafness identified (age range in months) |  | | |
| Aetiology of hearing loss (list causes) |  | | |
| Amplification used, i.e.. CI wearers n=6, HA = 7 |  | | |
| Amplification provided (age range in months) |  | | |
| Children with Additional needs | 1. Included 2. Excluded 3. 'No known additional needs' reported 4. Not reported 5. Other | | |
| Language(s) used by child (please list) |  | | |
| Child exposed to sign lang (any relevant information - parents are signers, child has a deaf instructor (i.e. native signer)? family had sign lessons? age of child when exposed to sign?) |  | | |
| **Caregiver characteristics** |  | | |
| Total sample size – parents (n) |  | |  |
| Sample size: Hearing parents of deaf children |  | |  |
| Sample size: Hearing parents of hearing children |  | |  |
| Age range of adult in years |  | |  |
| % of females (please do calculation) |  | | |
| Adult relationship to child |  | | |
| Ethnicity | 1. Ethnic majority group 2. Ethnic minority group 3. Mixed ethnic group 4. Not reported 5. Not clear 6. Other | | |
| Adult education level |  | | |
| Socio-eco status | 1. Mixed SES group 2. Low SES 3. Middle class SES 4. High SES 5. Not reported 6. Other | | |
| Language(s) used by adult |  | | |
|  |  | | |
| Language(s) used by adult with child |  | | |
| Adult’s prev. experience of deafness |  | | |
| **The methods and procedure of the study:** |  | | |
| Child Measures (list them, i.e. CDI, BPVS) |  | | |
| What specific aspects / behaviours are being assessed (list subtests, explicit behaviours /counts) |  | | |
| Child measures (tick) | 1. Pre-existing measure / validated measure 2. Novel measure devised for this study 3. Mixture of both validated and novel 4. None | | |
| Parent focused measure (i.e. parent stress, parent self efficacy) – do not report PCI procedure here). |  | | |
| Parent measures (tick) | 1. Pre-existing measure / validated measure 2. Novel measure devised for this study 3. Mixture of both validated and novel 4. None | | |
| Does this paper only use LENA data for PCI? | Y/N | | |
| Parent-Child Interaction measure(s) | Pre-existing measure / Validated measure i.e. EAS   1. Novel tool devised for this study 2. Coding 3. LENA | | |
|  |  | | |
| Video used | **Video - singular or series?**   1. No video 2. One off video – cross section 3. Video repeated over time – repeated measure | | |
| Software used for coding/analysis (i.e. ELAN, CLAN, INTERACT) |  | | |
| Length of interaction |  | | |
| Length of analysed section |  | | |
| Location of interaction (home, clinic, lab) |  | | |
| Interaction observed (i.e. snack time, 3 days of LENA recording) |  | | |
| Coding method explicitly shared (not what is being observed, but how) |  | | |
| Reliability procedures (i.e. time intervals watched) |  | |  |
| Blinding procedures |  | |  |
| **If intervention study:** |  | | |
| Named intervention |  | | |
| Style of intervention (group, 1:1 coaching, modelling, other) |  | | |
| Intervention length |  | | |
| Dose of intervention |  | | |
| Control or an alternative intervention mentioned? |  | | |
| **Results:** |  | | |
| Stats analysis used |  | | |
| Results on parent outcomes (T1/T2, means, median, range, scaled score, pre/post, change data) |  | | |
| Results on child outcomes (T1/T2, means, median, range, scaled score, pre/post, change data) |  | | |
| Results on PCI outcomes |  | | |
| **Confounding factors (identified by authors)** |  | | |
| **Limitations (identified by authors)** |  | | |
| Extractor general +/- comments |  | | |
